# Correction: Development of an enhanced analytical method utilizing pepper matrix as an analyte protectant for sensitive GC‒MS/MS detection of dimethipin in animal-based food products

**DOI:** 10.1371/journal.pone.0300856

**Published:** 2024-03-14

**Authors:** Jae-Han Shim, Md. Musfiqur Rahman, Tuba Esatbeyoglu, Fatih Oz, A. M. Abd El-Aty

The fifth author’s initials appear incorrectly in the citation. The correct citation is: Shim J-H, Rahman MM, Esatbeyoglu T, Oz F, Abd El-Aty AM (2023) Development of an enhanced analytical method utilizing pepper matrix as an analyte protectant for sensitive GC‒MS/MS detection of dimethipin in animal-based food products. PLoS ONE 18(12): e0295968. https://doi.org/10.1371/journal.pone.0295968.
