# Supplementary material for: Development of an enhanced analytical method utilizing pepper matrix as an analyte protectant for sensitive GC‒MS/MS detection of dimethipin in animal-based food products
Source: PLoS One. 2023 Dec 14;18(12):e0295968. doi: 10.1371/journal.pone.0295968 (PMC10721067; doi:10.1371/journal.pone.0295968)
Supplement: Attachment — Submitted filename: PLOS ONE reviewer-responses.docx [file pone.0295968.s001.docx]

**Journal requirements**:

When submitting your revision, we need you to address these additional requirements.

1. Please ensure that your manuscript meets PLOS ONE's style requirements, including those for file naming. The PLOS ONE style templates can be found at

<https://journals.plos.org/plosone/s/file?id=wjVg/PLOSOne_formatting_sample_main_body.pdf> and

<https://journals.plos.org/plosone/s/file?id=ba62/PLOSOne_formatting_sample_title_authors_affiliations.pdf>

Thank you. This has been done

2. We noticed you have some minor occurrence of overlapping text with the following previous publication(s), which needs to be addressed:

- <https://doi.org/10.1016/j.jchromb.2017.06.025>

Thank you. A slight overlap might occur due to similarities in authors' names, affiliations, chemicals and reagents, and common terminology, which is unavoidable.

In your revision ensure you cite all your sources (including your own works), and quote or rephrase any duplicated text outside the methods section. Further consideration is dependent on these concerns being addressed.

Done

3. Thank you for stating the following financial disclosure:

“The funders had no role in the study design, data collection and analysis, decision to publish, or preparation of the manuscript.”

At this time, please address the following queries:

a) Please clarify the sources of funding (financial or material support) for your study. List the grants or organizations that supported your study, including funding received from your institution.

b) State what role the funders took in the study. If the funders had no role in your study, please state: “The funders had no role in study design, data collection and analysis, decision to publish, or preparation of the manuscript.”

c) If any authors received a salary from any of your funders, please state which authors and which funders.

d) If you did not receive any funding for this study, please state: “The authors received no specific funding for this work.”

Please include your amended statements within your cover letter; we will change the online submission form on your behalf.

The authors received no specific funding for this work. We received only funding for the open access publication and stated within the manuscript as “The publication of this article was funded by the Open Access Fund of Leibniz Universität Hannover.”

4. In your Data Availability statement, you have not specified where the minimal data set underlying the results described in your manuscript can be found. PLOS defines a study's minimal data set as the underlying data used to reach the conclusions drawn in the manuscript and any additional data required to replicate the reported study findings in their entirety. All PLOS journals require that the minimal data set be made fully available. For more information about our data policy, please see <http://journals.plos.org/plosone/s/data-availability>.

"Upon resubmitting your revised manuscript, please upload your study’s minimal underlying data set as either Supporting Information files or to a stable, public repository and include the relevant URLs, DOIs, or accession numbers within your revised cover letter. For a list of acceptable repositories, please see <http://journals.plos.org/plosone/s/data-availability#loc-recommended-repositories>. Any potentially identifying patient information must be fully anonymized.

Important: If there are ethical or legal restrictions to sharing your data publicly, please explain these restrictions in detail. Please see our guidelines for more information on what we consider unacceptable restrictions to publicly sharing data: <http://journals.plos.org/plosone/s/data-availability#loc-unacceptable-data-access-restrictions>. Note that it is not acceptable for the authors to be the sole named individuals responsible for ensuring data access.

We will update your Data Availability statement to reflect the information you provide in your cover letter.

Thank you.

**Reviewers' comments**:

**Reviewer #1**: The manuscript titled: “Development of an enhanced analytical methods utilizing pepper matrix as an analyte protectant for sensitive GC‒MSMS detection of dimethipin in animal-based food products” is an interesting study with promising analytical applications. The text is composed in a fluent and accessible manner, making it suitable for a wide readership. However, the material and method section lacks some information and structure in the discussion section (major revisions). Please consider the comments and suggestions below to improve the manuscript:
We sincerely appreciate your thoughtful evaluation and constructive criticism of our manuscript titled "Development of an Enhanced Analytical Method Utilizing Pepper Matrix as an Analyte Protectant for Sensitive GC‒MS/MS Detection of Dimethipin in Animal-Based Food Products." Your insights have significantly contributed to enhancing the overall quality of our manuscript.

We have diligently addressed each of the comments and suggestions. Our aim was to offer a comprehensive and satisfactory response to your concerns, ensuring that the revised material and method section, as well as the discussion section, meet the standards expected for publication. We trust that the revisions made align with your expectations and contribute to the overall improvement of the manuscript.

Once again, we appreciate your meticulous review, and we remain open to any additional suggestions or feedback you may have. Your dedication to ensuring the rigor and clarity of scientific publications is highly valued.

**General comment**:

-the authors should explain the advantage of using the pepper leaf as an AP over other ones (e.g., 3-ethoxy-1,2-propanediol, D-sorbitol);

Thank you. This paragraph has been added to the text.

The choice of pepper leaf as an analyte protectant over alternatives, such as 3-ethoxy-1,2-propanediol or D-sorbitol, may offer several advantages:

1- Pepper leaf is a natural, plant-derived material, which might make it more compatible with certain analytes or matrices compared to synthetic compounds such as 3-ethoxy-1,2-propanediol.

2- The use of a natural analyte protectant could reduce interference in the analysis. Synthetic protectants might introduce additional compounds that could interfere with the detection or identification of the target analyte.

3- Plant-derived materials might have components that help in preserving sample integrity during the analysis. This is especially important when dealing with complex matrices, such as food products.

4- Pepper leaves, being a natural product, may be more cost-effective than synthetic alternatives.

5- The use of a natural product aligns with eco-friendly practices, which is increasingly important in analytical chemistry.

6- The specific chemical composition of pepper leaves might offer unique properties that enhance the stability of the analyte during analysis.

-Indicate how was the pepper leaf prepared and standardized over the different samples

This section has been conducted in accordance with our prior studies (Rahman et al., 2012; 2013; 2014)

- I suggest including statistical analyses (e.g. F test for the linearity results, and ANOVA to check the elution and differences between the QuEChERS methods);

Thank you for your suggestion. While we appreciate your emphasis on statistical analyses, we believe that it is not necessary to include them in this context. Our decision is based on the specific characteristics of our study and the nature of the data obtained.

- Discussion: please improve the connection between your results and previous works (e.g, lines 273-279, 292-295).

Thank you. This has been done

**Specific comments**:

Title: Keep the vertical bar between the MSMS as MS/MS;

This has been done

Keywords: use complementary words to the title; that will help to track your article in the search engines;

This has been amended as suggested.

Lines 52-60: Since the study is focused on this specific herbicide, it would be interesting to include information related to its use not only in the USA but in other locations (Europe, South America, Asia, and Africa). It would increase the importance of this study;

Thank you. Since the mid-1990s, the Americas have consistently held the position of the largest consumer of pesticides compared to other regions, surpassing Asia, Europe, Africa, and Oceania. As a result, the majority of reports tend to concentrate on the states rather than other regions.

Ref. Pesticides use and trade 1990–2021 FAOSTAT Analytical Brief 70

<https://www.fao.org/3/cc6958en/cc6958en.pdf>

Line 79: indicates the range of concentration units that can be found;

Thank you. There are currently no data available on the residual levels in products derived from livestock.

<https://www3.epa.gov/pesticides/chem_search/reg_actions/reregistration/fs_PC-118901_1-Sep-05.pdf>

Line 123: indicate between brackets the 2 articles. The same for the article in line 126;

Thank you. This has been added.

Line 147: please confirm if the mg of dimethipin used is 10.10 mg. In case the authors used the 10.10 mg, please correct the stock ppm values;

Yes, that is accurate. It is necessary to measure 10.10 mg from the dimethipin standard with a purity of 99%.

Lines 149-150: indicate here if this is a matrix-matched calibration curve;

Thank you. It is solvent calibration

Lines 151-152: please indicate for how long were these solutions stored;

Thank you. It is stored for a month.

Line 153: in the case of the spike samples, explain how they were done;

Thank you. This has been justified as follows: A total of 5 g of freeze-dried (chicken, pork, and beef) or fresh (egg and milk) samples was placed in a 50 mL Teflon centrifuge tube and spiked with 1 mL of 0.1 mg/kg (1 × limit of quantification [LOQ] level), 0.2 mg/kg (2 × LOQ level), or 1.0 mg/kg (10 × LOQ level).

Line 154: indicate if the matrices were fresh or freeze-dried

Thank you. The samples for chicken, pork, and beef were subjected to freeze-drying, while fresh samples were used for egg and milk.

Line 164: Please, indicate the final concentration factor for the samples

Thank you. The final concentration factor for the sample is 0.93. The concentration of AP was 30% (0.25 g/mL).

Line 171: indicate which type of liner was used in the injector port and the type of septum

Thank you. This information was added to the text. “The liner that was used in the injector port was Ultra inert liner, splitless single taper liner with glass wool (900 µL, 4 mm id (Part No. 5190-3163), whereas the septum was Inlet Septa: Agilent, Bleed/Temp optimized (BTO) nonstick 11 mm, 50/pk (Part No. 5183-4757)”.

Lines 185: Indicate how often were the liner, septum, and column changed (or cut in the case of the last one); also it would be important to explain how the authors control the quality of the injections without the presence of a deuterated compound;

Thank you. The frequency of change is twice a month for both the liner and septum, while for the column, it is once a month (1^st^ cutting).

Controlling the quality of injections in GC‒MS/MS without the presence of a deuterated compound involves several strategies:

1. Peak Shape and Resolution: Well-defined, symmetrical peaks with good resolution indicate a high-quality injection.

2. Baseline Stability: A stable baseline suggests proper injection and chromatographic conditions.

3. Retention Time Stability: Consistent retention times across injections indicate reproducibility.

4. Blank Runs: Injecting blanks (solvent only) to ensure that there is no contamination or interference. The baseline should remain stable.

5. Standard Solutions: Standard solutions were injected periodically to verify the instrument's sensitivity and linearity.

6. Matrix Effects: Evaluating the matrix effects by analyzing matrix-matched standards. This helps assess the impact of the sample matrix on the analytical signal.

7. Calibration standards: Calibration standards were injected regularly to ensure that the instrument response was consistent with the calibration curve.

8. Peak Identification: Confirming peak identity by comparing mass spectra and retention times with reference standards or library spectra.

Line 189: indicate in this section how the authors calculate the matrix effect;

Thank you. This has been added.

Line 192: indicate if the concentrations are expressed in FW;

Yes, it is expressed as fresh weight.

Line 205: in this section, the authors only mention the results in the solvent and egg matrix; please clarify if the optimizations were done only in those 2 matrices or for all of them;

Thank you. Yes, the process is conducted using solvent and egg matrix exclusively, serving as representatives for other matrices.

Lines 210-212: This sentence is confusing; please reformulate it. In addition, this information contradicts the information mentioned in lines 212-213;

Thank you. The highest was replaced with “satisfactory”.

Lines 229-257: If this step was performed before the elution step and the selection of the best QuEChERS method, please change the order of these sections;

Thank you. No, it was conducted after elution

Figure 3: indicate at which LOQ level were those tests performed; indicate also the value of the integrated areas;

Thank you. The caption has been revised to “**Figure 3.** GC‒MS/MS chromatograms of dimethipin in chicken (1), pork (2), beef (3), egg (4), and milk (5); a: standard in matrix (0.005 mg/kg), b: blank, c: recovery equivalent to 0.05 mg/kg, and d: market sample.

Lines 272-273: to improve the discussion section, please consider discussing the acquired LOQ values in terms of practicability to measure the herbicide in complex matrix samples;

Thank you. This has been amended as suggested.

Lines [273-279/292-295](tel:273-279/292-295): Please connect the information with the results of the article to improve the discussion section;

Thank you. This has been done

Lines 280-281: this sentence should be in the material e methods, in the method validation section; the same for 284-285;

Thank you. This has been amended as suggested.

Table 1: Please include the accuracy results too;

Thank you. The accuracy results are indicated by the recovery rates.

Line 302: indicate when were the samples bought; indicate here or in the method validation section which quality controls were used within samples and/or batch injections;

Thank you. The samples were acquired in 2019.

The QCs can be implemented through calibration standards, including blanks in each batch to monitor and correct for any contamination or background signals, and matrix-matched standards.

Line 314: please substitute GC/MS/MS with GC‒MS/MS.

Thank you. This has been amended.

**Response to Reviewer #2 comments**:

This manuscript falls under the PLOS ONE scope and presents findings of research title “Development of an Enhanced Analytical Method Utilizing Pepper Matrix as an Analyte Protectant for Sensitive GC‒MSMS Detection of Dimethipin in Animal-Based Food Products”. The manuscript consists of 24 pages, 3 figures and 1 table. The paper presents interesting results as well as an inquisitive and reliable interpretation of the research results. The topic original and relevant in the field of study. The Abstract provides the highlights of the key contents of the main text. The Introduction provides enough background information to justify the study. The Results are consistent with the declared methodology, presented clearly enough, supported by the figures and tables. Researchers devised and validated an analytical method using GC‒MS/MS detection of dimethipin and concluded that this method effectively addresses the challenge of analyzing dimethipin and can be used as a routine monitoring tool for dimethipin across various matrices. The methodology adequately described and conclusion consistent with the evidence and arguments presented. The references are appropriate and relevant to the research. However, minor typographical and grammatical errors need addressing.

Thank you. We have checked the text once again for grammar and syntax errors.
