# Supplementary material for: Development of an enhanced analytical method utilizing pepper matrix as an analyte protectant for sensitive GC‒MS/MS detection of dimethipin in animal-based food products
Source: PLoS One. 2023 Dec 14;18(12):e0295968. doi: 10.1371/journal.pone.0295968 (PMC10721067; doi:10.1371/journal.pone.0295968)
Supplement: Attachment — Submitted filename: PLOS ONE - Response -R2.docx [file pone.0295968.s002.docx]

**Response to the journal and reviewer’s comments**

**Journal Requirements**

Please review your reference list to ensure that it is complete and correct. If you have cited papers that have been retracted, please include the rationale for doing so in the manuscript text, or remove these references and replace them with relevant current references. Any changes to the reference list should be mentioned in the rebuttal letter that accompanies your revised manuscript. If you need to cite a retracted article, indicate the article’s retracted status in the References list and include a citation and full reference for the retraction notice.

**Response**: Thank you for your thorough review of our manuscript and the reference list. We have carefully examined all cited references, and we can confirm that none of them were taken from retracted articles. The reference list has been reviewed for completeness and accuracy, and we believe it accurately represents the current state of the literature related to our work.

**Reviewer # 1 comments**:

Reviewer #1: The authors have responded to all queries raised by the reviewer, enhancing the overall quality of the manuscript. Prior to acceptance, kindly incorporate some of the provided responses (indicated as R below) to elucidate these aspects. This clarification aims not only to address the concerns raised by the reviewer but also to provide enhanced clarity for all readers.

Original:Lines 52-60: Since the study is focused on this specific herbicide, it would interesting to include information related to its use not only in the USA but in other locations (Europe, South America, Asia, and Africa). It would increase the importance of this study;
R: Thank you. Since the mid-1990s, the Americas have consistently held the position of the largest consumer of pesticides compared to other regions, surpassing Asia, Europe, Africa, and Oceania. As a result, the majority of reports tend to concentrate on the states rather than other regions. Ref. Pesticides use and trade 1990–2021 FAOSTAT Analytical Brief 70 <https://www.fao.org/3/cc6958en/cc6958en.pdf>
-Please add that information to the manuscript

Original:Line 164: Please, indicate the final concentration factor for the samples
R: Thank you. The final concentration factor for the sample is 0.93. The concentration of AP was 30% (0.25 g/mL).
-Please indicate that information in the manuscript

Original:Lines 185: Indicate how often were the liner, septum, and column changed (or cut in the case of the last one); also it would be important to explain how the authors control the quality of the injections without the presence of a deuterated compound;
R: Thank you. The frequency of change is twice a month for both the liner and septum, while for the column, it is once a month (1st cutting).
-Please indicate that information in the manuscript
R: Controlling the quality of injections in GC‒MS/MS without the presence of a deuterated compound involves several strategies:…..
-Use that information in the discussion to support the quality of your analyses

Original:Line 192: indicate if the concentrations are expressed in FW;
R: Yes, it is expressed as fresh weight
-Please add that information in the manuscript.

Original:Line 302: indicate when were the samples bought; indicate here or in the method validation section which quality controls were used within samples and/or batch injections;
R: 1)Thank you. The samples were acquired in 2019.
R: 2)The QCs can be implemented through calibration standards, including blanks in each batch to monitor and correct for any contamination or background signals, and matrix matched standards.
-1) please indicate that information in the method application section
-2) please indicate that information in the material and methods section

**Response:** Thank you to the diligent reviewer for the feedback. We have carefully reviewed the comments and suggestions and have incorporated the necessary additions and clarifications as indicated by "R" in the revised manuscript. We believe that these changes enhance the overall clarity of the manuscript for all readers, as denoted by the esteemed reviewer.
